# Validierung der deutschen Version des Fragebogens zur dermatologischen Lebensqualität von Säuglingen und Kleinkindern (InToDermQoL)

**DOI:** 10.1111/ddg.16008_g

**Published:** 2026-09-08

**Authors:** Juliane Traxler, Neuza da Silva Burger, Matthias Augustin, Hagen Ott, Sanna Hoffmann, Regina Fölster‐Holst, Maria Baumeister, Petra Staubach, Rachel Sommer

**Affiliations:** ^1^ Institut für Versorgungsforschung in der Dermatologie und bei Pflegeberufen (IVDP) Universitätsklinikum Hamburg‐Eppendorf (UKE), Hamburg; ^2^ Pädiatrische Dermatologie und Allergologie Kinder‐ und Jugendkrankenhaus Auf der Bult, Hannover; ^3^ Klinik für Dermatologie Venerologie und Allergologie Universitätsklinikum Schleswig‐Holstein Campus Kiel, Kiel; ^4^ Fachklinik Gaißach, Gaißach; ^5^ Hautklinik und Poliklinik Universitätsmedizin Mainz

**Keywords:** Atopische Dermatitis, Kleinkinder und Säuglinge, Lebensqualität, patientenberichteter Endpunkt, pädiatrische Dermatologie, psychometrische Studie, Atopic dermatitis, outcome measure, pediatric dermatology, psychometrics, quality of life, toddlers and infants

## Abstract

**Hintergrund und Zielsetzung:**

Hauterkrankungen können die Lebensqualität (*quality of life*; QoL) von pädiatrischen Patienten und ihren Familien erheblich beeinträchtigen. Der Fragebogen zur Lebensqualität in der Dermatologie bei Säuglingen und Kleinkindern (*Infants and Toddlers Dermatology Quality of Life questionnaire*; InToDermQoL) ist das erste hautgenerische Instrument zur Bewertung der QoL bei Kindern ≤ 4 Jahren, wie sie von ihren Bezugspersonen angegeben wird. Ziel dieser Studie war es, die deutsche Version des InToDermQoL psychometrisch zu validieren.

**Patienten und Methodik:**

Die psychometrischen Eigenschaften des InToDermQoL wurden an einer Stichprobe von 148 Kindern mit verschiedenen Hauterkrankungen untersucht (Altersgruppen [n]: < 1 Jahr [44]; 1–2 Jahre [68]; 3–4 Jahre [36]).

**Ergebnisse:**

Die explorative Faktorenanalyse ergab eine eindimensionale Struktur der Version für Kinder < 1 Jahr und eine Zwei‐ beziehungsweise Dreifaktorenstruktur für die beiden älteren Altersgruppen. Die interne Konsistenz war ausgezeichnet (Cronbachs Alpha: 0,904–0,922). Die Test‐Retest‐Reliabilität über zwei Wochen war gut, mit Ausnahme der 1‐ bis 2‐jährigen Kinder (Intraklassenkorrelationskoeffizient; ICC = 0,430). Der InToDermQoL zeigte eine gute konvergente Validität mit anderen QoL‐Fragebögen.

**Schlussfolgerungen:**

Der deutsche InToDermQoL ist ein valides und zuverlässiges Instrument zur Messung der Lebensqualität von Kindern ≤ 4 Jahren mit Hauterkrankungen. Der Einsatz des Instruments in Forschung und klinischer Praxis wird zu besserem Verständnis der Auswirkungen pädiatrischer Dermatosen beitragen und die Versorgung und Unterstützung betroffener Familien optimieren.

## EINFÜHRUNG

Hauterkrankungen treten in der frühen Kindheit sehr häufig auf: Allein die atopische Dermatitis (AD) hat in Deutschland eine geschätzte Prävalenz von 17 % bei Säuglingen im Alter von 0 bis 2 Jahren und von 13 % bei Kindern im Alter von 3 bis 6 Jahren.^1^ Pädiatrische Dermatosen verursachen jedoch nicht nur körperliche Symptome, sondern können auch die allgemeine Funktionsfähigkeit der Familie,^2^, ^3^ die psychosoziale und körperliche Entwicklung des Kindes, sein Selbstbild sowie seine gesundheitsbezogene Lebensqualität (HRQoL) erheblich beeinträchtigen.^4^, ^5^, ^6^, ^7^, ^8^ Daher besteht sowohl in der klinischen Versorgung als auch in Studien unter realen Bedingungen ein großer Bedarf, die HRQoL junger Patienten mit Hauterkrankungen zu erfassen. Das Verständnis der Krankheitslast sowie der Bedürfnisse und Anliegen der einzelnen Patienten und ihrer Bezugspersonen im Rahmen eines familienzentrierten Ansatzes würde es den Gesundheitsdienstleistern ermöglichen, die Behandlung individuell anzupassen und die Versorgung zu optimieren. Dies würde auch zu einer stärker personen‐ und menschenzentrierten Versorgung dieser jungen Patienten und ihrer Familien beitragen.^9^, ^10^, ^11^

Allerdings gibt es nur sehr wenige Studien, die sich mit der HRQoL bei Kleinkindern/Vorschulkindern mit Hauterkrankungen befassen, was vor allem auf den Mangel an validen Instrumenten zurückzuführen ist. Die Entwicklung solcher Instrumente wird durch die raschen Entwicklungsveränderungen in den ersten Lebensjahren und die Tatsache erschwert, dass anstelle einer direkten Befragung eine stellvertretende Beurteilung durch die Eltern oder andere Bezugspersonen erforderlich ist. Darüber hinaus ist atopische Dermatitis zwar weit verbreitet, andere Hauterkrankungen (zum Beispiel Psoriasis, Pityriasis rosea) sind jedoch seltener und machen die Entwicklung krankheitsspezifischer Fragebögen unpraktikabel.

Um diese Lücke zu schließen, wurde der Fragebogen *Infants and Toddlers Dermatology Quality of Life* (InToDermQoL) als hautgenerisches Proxy‐Maß für die HRQoL,^12^ gemäß der von den europäischen KIDSCREEN/DISABKIDS‐Gruppen vorgeschlagenen Methodik für die Entwicklung kulturübergreifender Instrumente entwickelt.^13^ Die drei altersspezifischen Versionen des Fragebogens mit jeweils 10, 12 und 15 Items wurden bereits in einigen Sprachversionen psychometrisch getestet und bestätigten eine hohe interne Konsistenz, eine ausgezeichnete Test‐Retest‐Reliabilität und eine gute konvergente Validität.^14^ Darüber hinaus wurden die Responsivität und der minimal klinisch relevante Unterschied des Instruments bestätigt.^15^, ^16^ Obwohl Deutschland an der Pilotphase der Instrumentenentwicklung teilgenommen hat,^12^ wurde die deutsche Version bisher nicht validiert. Das aktuelle Projekt hatte zum Ziel, den deutschen InToDermQoL gemäß den internationalen Richtlinien der US‐amerikanischen *Food and Drug Administration* zur Bewertung der Messungseigenschaften von PRO‐Messinstrumenten,^17^ für den Einsatz in Forschung und klinischer Praxis psychometrisch zu validieren.

## MATERIALIEN UND METHODIK

Diese Studie hatte ein querschnittsorientiertes Beobachtungsdesign und wurde gemäß den Richtlinien für internationale Feldtests des InToDermQoL‐Fragebogens und den allgemeinen Empfehlungen für die Messung der HRQoL durchgeführt.^13^, ^18^, ^19^ Die Datenerhebung fand zwischen August 2021 und April 2024 statt. Die Studie wurde in Übereinstimmung mit der Helsinki‐Erklärung durchgeführt und von der Ethikkommission des Universitätsklinikums Hamburg‐Eppendorf genehmigt (Genehmigungsnummer: LPEK‐0304).

### Teilnehmer

An der Umfrage wurden Bezugspersonen von Säuglingen und Kleinkindern im Alter von 0 bis 4 Jahren mit einer Hauterkrankung jeglicher Art eingeladen. Die Teilnehmer wurden in sechs deutschen Hautkliniken und Kinderkliniken rekrutiert und gaben vor Beginn der Studie ihre schriftliche Einverständniserklärung ab. Die Teilnahmebedingungen wurden vom behandelnden Arzt überprüft und umfassten: Betreuung eines Kindes im Alter von 0 bis 4 Jahren mit einer diagnostizierten Hauterkrankung jeglicher Art; Fähigkeit zur selbstständigen Einwilligung sowie ausreichende Deutschkenntnisse in Wort und Schrift.

### Endpunkte

Die Daten wurden auf Papier erhoben. Es wurden die deutschen Versionen der folgenden Fragebögen verwendet.

### Arzt‐berichtete Endpunkte

*Globale klinische Bewertung (Global Clinical Assessment; GCA)*. Die GCA ist eine Ein‐Punkt‐Skala zur Bewertung des Schweregrads einer Hauterkrankung auf einer 5‐Punkte‐Likert‐Skala von 0 (nicht ausgeprägt) bis 4 (sehr schwer).

*SCORing Atopic Dermatitis (SCORAD)*.^19^ Der SCORAD bewertet Ausmaß und Schweregrad der atopischen Dermatitis. Es wird eine Gesamtpunktzahl berechnet, die zwischen 0 (minimaler Schweregrad) und 103 (maximaler Schweregrad) liegen kann. SCORAD‐Werte < 25 gelten als leichte atopische Dermatitis, Werte zwischen 25 und 59 als mittelschwere atopische Dermatitis und Werte ≥ 60 als schwere atopische Dermatitis. Die psychometrischen Eigenschaften dieses Instruments bei Säuglingen und Kleinkindern sind gut.^20^ Dieser Fragebogen wurde nur für Patienten mit atopischer Dermatitis ausgefüllt.

### Von Bezugspersonen berichtete Endpunkte

*InToDermQoL*.^12^ Die deutsche Pilotversion des InToDermQoL,^12^ wurde auf der Grundlage der endgültigen englischen Fassung,^14^ überarbeitet und umfasst zehn Items für Eltern/Bezugspersonen von Kindern unter einem Jahr, zwei zusätzliche Items für Eltern/Bezugspersonen von Kindern im Alter von 1 bis 2 Jahren und drei weitere Items für Eltern/Bezugspersonen von Kindern im Alter von 3 bis 4 Jahren. Die *recall period* beträgt eine Woche und die Items sind auf einer 4‐Punkte‐Skala von 0 (gar nicht) bis 3 (sehr) zu beantworten. Der Gesamtscore wird durch Addition aller Items berechnet, wobei niedrigste Score 0 und der höchste Score 30 für Kinder unter 1 Jahr, 36 für Kinder zwischen 1 und 3 Jahren und 45 für Kinder zwischen 3 und 4 Jahren beträgt, wobei höhere Werte auf stärkere Beeinträchtigungen der HRQoL hinweisen. Zudem können die Items in den Versionen für Kinder unter 3 Jahren in zwei Bereiche (Symptome und Alltagsaktivitäten) und für Kinder über 3 Jahren in drei Bereiche (Symptome, Alltagsaktivitäten und Beziehungen zu anderen Menschen) gruppiert werden, obwohl die faktorielle Struktur des Fragebogens bisher nicht getestet wurde.

*Infants' Dermatitis Quality of Life Index (IDQOL)*.^21^ Der IDQOL ist ein krankheitsspezifischer HRQoL‐Fragebogen, der von Eltern von Kleinkindern bis zu 4 Jahren mit atopischer Dermatitis ausgefüllt wird. Der Fragebogen enthält zehn Fragen zu Symptomen und Schwierigkeiten in Bezug auf Stimmung, Schlaf, Spiel, Familienaktivitäten, Mahlzeiten, Behandlungen, Ankleiden und Baden. Die Items werden auf einer Likert‐Skala von 0 bis 3 bewertet; aus der Summe der zehn Items (0–30) kann eine Gesamtpunktzahl berechnet werden, wobei höhere Werte auf eine stärkere Beeinträchtigung der HRQoL hinweisen.

*Pediatric Quality of Life Inventory (PedsQL^TM^)*.^22^, ^23^ Der PedsQL^TM^ wurde als generisches HRQoL‐Instrument entwickelt, um die Wahrnehmung der Eltern hinsichtlich der allgemeinen HRQoL ihres Kindes zu bewerten. Der PedsQL^TM^ Infant Scales umfasst zwei altersgerechte Versionen: eine für Säuglinge im Alter von 1 bis 12 Monaten (36 Items) und eine für Kleinkinder im Alter von 13 bis 24 Monaten (45 Items). Die Items sind auf einer 5‐Punkte‐Likert‐Skala von 0 (nie ein Problem) bis 4 (fast immer ein Problem) zu beantworten. Die Items werden umkodiert und linear auf eine Skala von 0 bis 100 transformiert, wobei höhere Werte eine bessere HRQoL anzeigen. Die deutsche Version hat gute psychometrische Eigenschaften gezeigt.^24^

### Vorgehen

Der behandelnde Arzt berichtete soziodemografische (Alter, Geschlecht, Größe, Gewicht) und klinische Daten (Hauterkrankung, aktuelle Behandlung, Begleiterkrankungen, betroffene Körperoberfläche [0–100 %], Schweregrad der Erkrankung [GCA, SCORAD]) des Patienten. Zu Beginn der Studie (T0) gaben die Bezugspersonen soziodemografische Informationen über sich selbst (Alter, Beziehung zum Kind) und das Kind (Alter, Geschlecht, Hauptbezugsperson), klinische Informationen über das Kind (Hauterkrankung, Alter bei der Diagnose, Allergien und Überempfindlichkeiten, aktuelle Symptome und Medikamente, Ernährung) sowie einige Fragebögen, wie oben beschrieben, an. Ein Viertel der Befragten wurde gebeten, 14 Tage später (T1) eine verkürzte Version des Fragebogens mit soziodemografischen und klinischen Fragen sowie den InToDermQoL auszufüllen und per Umschlag an das koordinierende Studienzentrum zurückzusenden.

### Statistische Analysen

Die statistischen Analysen wurden mit dem Statistical Package for the Social Sciences (SPSS v.27.0; IBM Corp., Armonk, NY) durchgeführt. Das Signifikanzniveau wurde auf einen p‐Wert ≤ 0,05 festgelegt. Es wurden deskriptive Statistiken zu soziodemografischen und klinischen Merkmalen für die Gesamtstichprobe und für die drei Altersgruppen < 1 Jahr, zwischen 1 und 3 Jahren und zwischen 3 und 4 Jahren erstellt.

Die Verteilungsmerkmale der *Items*, einschließlich Boden‐ und Deckeneffekte, Schiefe und Kurtosis, wurden überprüft. Die strukturelle Validität des deutschen InToDermQoL wurde mittels explorativer Faktorenanalyse (EFA) untersucht. Der Bartlett‐Test auf Sphärizität und die KMO‐Statistik,^25^ wurden verwendet, um sicherzustellen, dass die Korrelationsmatrix nicht zufällig war. Die Faktorenanalyse wurde mittels Hauptkomponentenanalyse durchgeführt. Da davon ausgegangen wurde, dass die Faktoren korreliert sind, wurde eine Oblimin‐Rotation verwendet. Musterkoeffizienten ≥ 0,3 wurden als salient erachtet. Kreuzladungen (Ladungen, die auf mehr als einem Faktor salient sind) wurden verworfen, um eine einfache Struktur zu erreichen.^26^ Faktoren mit mindestens drei salienten Ladungen, einer internen Konsistenz von *α* ≥ 0,7 und theoretischer Aussagekraft wurden als adäquat angesehen.

Die interne Konsistenz des InToDermQoL‐Fragebogens wurde anhand der Cronbach‐Alpha‐Koeffizienten bewertet. Um die zweiwöchige Test‐Retest‐Reliabilität zu bestimmen, wurden Intraklassenkorrelationskoeffizienten (ICC) in einem absoluten Übereinstimmungsmodell mit zwei gemischten Effekten berechnet.^27^ Sowohl für die interne Konsistenz als auch für die Test‐Retest‐Reliabilität wurden Koeffizienten ≥ 0,50, ≥ 0,75 und ≥ 0,90 als moderate, gute bzw. ausgezeichnete Reliabilität angesehen. Um zu beurteilen, ob sich die Stichprobe, die T1 ausgefüllt hat, von der Gesamtstichprobe unterscheidet, wurden unabhängige t‐Tests zu den soziodemografischen (Alter, Geschlecht) und klinischen Merkmalen (SCORAD) des Kindes, dem Alter der Pflegeperson, die den Fragebogen ausgefüllt hat, und den InToDermQoL‐Werten zu T0 durchgeführt. Die *Standardmessfehler* (*standard error of measurement*; SEM) für die drei Altersversionen wurden als $\mathrm{SEM}=\frac{SD_{Differenz}}{\surd 2}$ berechnet.^28^, ^29^ Minimal nachweisbare Veränderungen (*minimal detectable change*; MDC) können sowohl für einzelne Probanden als auch für Gruppenvergleiche berechnet werden, wobei erstere für die klinische Praxis zur Bestimmung individueller Veränderungen informativ sind, letztere hingegen für die Interpretation von Durchschnittswerten von Gruppen, zum Beispiel im Forschungskontext.^29^ Die MDC‐Werte wurden mit einem Konfidenzintervall von 95 % nach der Formel MDC_individuell_ = SEM × 1,96 × √2 und $\mathrm{MD}C_{\mathrm{Gruppe}}=\frac{MDC_{\mathrm{individell}}}{\sqrt{n}}$ berechnet.^29^, ^30^

Die Konstruktvalidität wurde anhand der konvergenten Validität und der Validität bekannter Gruppen bestimmt. Die konvergente Validität wurde mit Pearson‐Korrelationskoeffizienten zwischen dem hautgenerischen InToDermQoL und zuvor validierten generischen Instrumenten (PedsQL^TM^ Generic Core Module) untersucht. Für die Untergruppe der Patienten mit atopischer Dermatitis wurde der InToDermQoL auch mit den krankheitsspezifischen IDQOL und SCORAD korreliert. Die Validität bekannter Gruppen wurde mittels ANOVAs getestet, wobei die InToDermQoL‐Werte über die Schweregrade der GCA hinweg verglichen wurden. Die folgenden a priori definierten Hypothesen wurden für jede der drei Altersgruppen getestet, von denen mindestens 75 % erfüllt sein mussten, um die Konstruktvalidität zu bestätigen:

1. Der InToDermQoL und der PedsQL zeigen eine moderate negative Korrelation,^14^ [r = –0,30 bis –0,49)].^31^
2. Der InToDermQoL und der IDQOL zeigen eine moderate bis hohe positive Korrelation (r ≥ 0,30).^31^
3. Der InToDermQoL und der SCORAD zeigen eine geringe bis moderate positive Korrelation (r = 0,10–0,49).^31^
4. Kinder mit mittelschwerer bis schwerer Erkrankung (GCA ≥ 2) haben höhere InToDermQoL‐Werte als Kinder ohne oder mit leichter Erkrankung (GCA < 2).

## ERGEBNISSE

Insgesamt wurden 148 Kinder (56,1 % männlich) mit einem Durchschnittsalter von 1,85 Jahren (Standardabweichung [SD] = 1,1) in die Studie aufgenommen. Demografische und klinische Informationen zu den teilnehmenden Kindern und Befragten sind in Tabelle 1 aufgeführt.

**TABELLE 1:** Demografische Daten und klinische Merkmale (Mittelwert ± SD) der eingeschlossenen Kinder und Bezugspersonen in der Gesamtstichprobe und in den verschiedenen Altersgruppen.

		Gesamt (n = 148)	< 1 Jahr (n = 44)	1–2 Jahre (n = 68)	3–4 Jahre (n = 36)
***Kind***					
Geschlecht (%)	Weiblich	65 (43,91)	21 (47,73)	23 (33,82)	21 (58,33)
	Männlich	83 (56,08)	23 (52,27)	45 (66,18)	15 (41,67)
Alter (in Jahren)		1,85 (± 1,12)	0,58 (± 0,54)	1,91 (± 0,56)	3,34 (± 0,47)
Alter bei Erstauftreten (in Jahren)		0,57 (± 0,59)	0,23 (± 0,16)	0,64 (± 0,55)	0,84 (± 0,79)
Dauer der Schübe (in Monaten)		2,28 (± 1,66)	2,66 (± 1,75)	2,23 (± 1,62)	2,00 (± 1,60)
Längste Phase ohne Auftreten (in Monaten)		2,38 (± 2,62)	0,83 (± 1,48)	2,56 (± 2,88)	2,99 (± 2,40)
Therapie ( %)^*^	Keine	4 (16,2)	2 (4,5)	1 (1,5)	1 (2,8)
	Topisch	130 (87,8)	34 (77,3)	61 (89,7)	35 (97,2)
	Systemisch	18 (12,2)	7 (15,9)	11 (16,2)	0
	Andere	17 (11,5)	10 (22,7)	5 (7,4)	2 (5,6)
GCA (%)	1	5 (3,4)	2 (4,5)	2 (2,9)	1 (2,8)
	2	52 (35,1)	15 (34,1)	28 (45,9)	11 (30,6)
	3	61 (41,3)	22 (50,0)	24 (39,3)	13 (36,1)
	4	18 (12,2)	5 (11,4)	7 (10,3)	6 (16,7)
BSA (in %)		18,06 (± 19,35)	15,37 (± 16,52)	19,30 (± 20,96)	19,37 (± 19,90)
SCORAD^**^		31,6 (± 16,00)	35,23 (± 13,60)	30,16 (± 17,52)	30,66 (± 15,33)
***Diagnose***					
Nicht infektiös	Atopische Dermatitis		31	55	32
	Hämangiom		10	1	1
	Becker Naevus		1		
	Lymphangiom		1		
	Neurofibromatose		1		
	Seborrhoische Dermatitis		1		
	Windeldermatitis			2	1
	Ichthyosis vulgaris			1	1
	Alopecia areata			1	
	Kutane Mastozytose			1	
	Eczema infantum			1	
	Juveniles Xanthogranuloma			1	
	Keratosis pilaris			1	
	Milium			1	
	Onychodystrophie			1	
	Lichen vidal				1
	Psoriasis				1
	Tinea amiantacea				1
	Naevus		1		
Bakterielle Infektion	Superinfektion		2	1	
	Impetigo			1	
	Panaritium			1	
Parasiteninfektion	Scabies			2	
Pilzinfektion	Tinea capitis			1	
Virusinfektion	Herpes simplex			1	
	Urticaria exanthema				1
**Bezugsperson**					
Mutter (%)		131 (88,5)	37 (84,1)	62 (91,2)	32 (88,9)
Vater (%)		14 (9,5)	6 (13,6)	4 (5,9)	4 (11,1)
Beide Eltern (%)		1 (0,7)	1 (2,3)	–	–
Andere (%)		1 (0,7)	–	1 (1,5)	–
Alter (in Jahren)		33,59 (± 5,48)	31,51 (± 4,45)	33,59 (± 5,39)	36,06 (± 5,84)

*Abk*.: GCA, *Global Clinical Assessment* (1, sehr leicht–leicht; 2, leicht–moderat; 3, moderat–schwer; 4, schwer–sehr schwer); BSA, *Body Surface Area*; SCORAD, *SCORing Atopic Dermatitis*

* 14 Kinder erhielten mehr als eine Art von Behandlung

** n = 109

In keiner der Altersgruppen wurden Deckeneffekte beobachtet; in der jüngsten Altersgruppe war jedoch ein Bodeneffekt zu verzeichnen (27,3 %) (Tabelle 2). Schiefe und Kurtosis lagen im akzeptablen Bereich (± 1,5).^32^ Die Item‐Gesamt‐Korrelationen lagen meist über 0,50, wobei 1–2 Items pro Altersversion nur moderate Korrelationen aufwiesen. Da die Streichung dieser Items den Cronbach‐Alpha‐Wert nicht wesentlich erhöht hätte, wurden diese Items beibehalten. Informationen zu IDQOL und PedsQL^TM^ sowie detaillierte psychometrische Ergebnisse des InToDermQoL sind in den Online‐Supplements S1 und S2 zu finden.

**TABELLE 2:** Beschreibende Statistik des InToDermQoL für die Gesamtstichprobe und die Altersgruppen zu Beginn und bei der Nachuntersuchung (T1).

Altersgruppe	Item	MW (SD)	Bereich	Schiefe	Kurtosis	Bodeneffekte	Deckeneffekte	Cronbachs Alpha	Item‐Gesamt‐Korrelation	Cronbachs Alpha, wenn Item gelöscht
< 1 Jahr	Gesamtskala	8,57 (7,26)	0–26	0,197	−1,081	27,3 %	0 %	0,912		
	1	1,58 (1,36)							0,824	0,894
	2	1,05 (1,04)							0,765	0,897
	3	0,88 (0,94)							0,766	0,898
	4	1,20 (1,16)							0,887	0,888
	5	0,80 (0,97)							0,812	0,895
	6	0,53 (0,93)							0,472	0,914
	7	0,70 (0,97)							0,686	0,902
	8	0,38 (0,81)							0,416	0,916
	9	0,38 (0,74)							0,532	0,910
	10	0,50 (0,75)							0,627	0,906
1–2 Jahre	Gesamtskala	11,62 (8,31)	0–30	0,573	−0,574	5,9 %	0 %	0,904		
	1	1,87 (1,09)							0,623	0,896
	2	1,23 (1,09)							0,607	0,897
	3	1,15 (1,03)							0,719	0,891
	4	1,44 (1,16)							0,760	0,889
	5	1,03 (1,10)							0,836	0,885
	6	0,70 (0,97)							0,697	0,893
	7	0,59 (0,84)							0,591	0,898
	8	0,49 (0,87)							0,569	0,899
	9	0,36 (0,66)							0,601	0,899
	10	0,84 (1,04)							0,551	0,900
	11	0,97 (1,10)							0,635	0,896
	12	0,69 (1,08)							0,404	0,907

Altersgruppe	Item	MW (SD)	Bereich	Schiefe	Kurtosis	Bodeneffekte	Deckeneffekte	Cronbachs Alpha	Item‐Gesamt‐Korrelation	Cronbachs Alpha, wenn Item gelöscht
3–4 Jahre	Gesamtskala	15,03 (9,39)	2–38	0,941	0,001	2,8 %	0 %	0,922		
	1	2,26 (0,86)							0,544	0,920
	2	1,70 (0,91)							0,552	0,920
	3	1,44 (0,93)							0,532	0,921
	4	1,52 (0,89)							0,640	0,917
	5	1,07 (0,96)							0,767	0,913
	6	0,93 (1,21)							0,693	0,916
	7	0,70 (0,95)							0,823	0,911
	8	0,48 (0,85)							0,726	0,915
	9	0,63 (1,08)							0,760	0,913
	10	1,11 (1,01)							0,643	0,917
	11	1,00 (0,92)							0,688	0,916
	12	0,78 (1,05)							0,708	0,915
	13	0,44 (0,70)							0,582	0,919
	14	0,59 (0,69)							0,534	0,920
	15	0,15 (0,36)							0,380	0,924

**T1**										
< 1 Jahr	Gesamtskala	9,91 (6,47)	0–21	0,459	−0,733	9,1 %	0 %	0,876		
1–2 Jahre	Gesamtskala	6,72 (5,17)	0–18	0,580	−0,543	6,9 %	0 %	0,897		
3–4 Jahre	Gesamtskala	9,15 (7,96)	2–26	1,249	0,485	7,5 %	0 %	0,890		

*Abk*.: MW, Mittelwert; Standardabweichung, SD

### Strukturelle Validität

Die EFA ergab, dass die Version für Säuglinge (< 1 Jahr) eindimensional ist und 61 % der Gesamtvarianz mit einem Cronbachs Alpha von 0,917 erklärt. Die Version für 1‐ bis 2‐jährige Kinder schien zwei Faktoren zu umfassen: „Symptome“ (5 Items) und „Alltagsaktivitäten“ (7 Items). Schließlich wurde eine 3‐Faktoren‐Lösung als die am besten geeignete strukturelle Darstellung der Version für 3‐ bis 4‐Jährige angesehen, mit den Faktoren „Alltagsaktivitäten“ (6 Items), „Symptome“ (4 Items) und „Beziehungen zu anderen“ (5 Items). Die Faktorladungen und ihre Merkmale für die beiden älteren Altersgruppen sind in den Tabellen 3 und 4 dargestellt, eine ausführliche Beschreibung der EFA findet sich in Online‐Supplement S2.

**TABELLE 3:** Faktorladungen, erklärte Gesamtvarianz und Cronbachs Alpha der 2‐Faktoren‐Lösung für die deutsche Version des InToDermQoL bei 1–2‐jährigen Kindern (n = 68).

	Faktor	
	1	2
Ihr Kind hat aufgrund seiner Hautkrankheit geblutet (von verletzter Haut und / oder Schleimhaut)	**0,906**	
Ihr Kind hatte Schlafprobleme aufgrund seiner Hautkrankheit	**0,805**	
Ihr Kind hatte Schmerzen aufgrund seiner Hautkrankheit	**0,801**	
Ihr Kind hatte Juckreiz und hat sich aufgrund seiner Hautkrankheit gekratzt	**0,720**	
Ihr Kind hatte Stimmungsveränderungen aufgrund seiner Hautkrankheit	**0,720**	
Ihr Kind hatte Einschränkungen und Grenzen aufgrund seiner Hautkrankheit (sozial, Ernährung, körperliche Aktivität und Sport, Haustiere, usw.)		**0,771**
Ihr Kind hatte Probleme beim Essen aufgrund seiner Hautkrankheit		**0,708**
Ihr Kind war müde oder erschöpft aufgrund seiner Hautkrankheit		**0,662**
Ihr Kind hatte Probleme bei körperlicher Aktivität (Bewegungen für Säuglinge; Laufen, Krabbeln, usw. für Kleinkinder) aufgrund seiner Hautkrankheit		**0,657**
Ihr Kind hatte Probleme beim Baden aufgrund seiner Hautkrankheit	0,331	**0,529**
Ihr Kind hatte Probleme mit der Behandlung (z. B. Behandlung zu Hause, Bandagieren, Hautpflege, usw.) aufgrund seiner Hautkrankheit		**0,433**
Ihr Kind hatte Probleme beim An‐ und Ausziehen (Reizung der betroffenen Stellen, Schmerzen) aufgrund seiner Hautkrankheit	0,363	**0,364**
**Erklärte Gesamtvarianz (in %)**	49,9	13,8
**Cronbachs Alpha**	0,910	0,843

Faktor 1: “Symptome”, Faktor 2: “Alltagsaktivitäten”.

**TABELLE 4:** Faktorladungen, erklärte Gesamtvarianz und Cronbachs Alpha der 3‐Faktoren‐Lösung für die deutsche Version des InToDermQoL bei 3–4‐jährigen Kindern (n = 36).

	Faktor		
	1	2	3
Ihr Kind hatte Probleme bei körperlicher Aktivität (Bewegungen für Säuglinge; Laufen, Krabbeln, usw. für Kleinkinder) aufgrund seiner Hautkrankheit	**0,924**		
Ihr Kind hatte Probleme beim Essen aufgrund seiner Hautkrankheit	**0,812**		
Ihr Kind hatte Einschränkungen und Grenzen aufgrund seiner Hautkrankheit (sozial, Ernährung, körperliche Aktivität und Sport, Haustiere, usw.)	**0,807**		
Ihr Kind hatte Probleme beim Baden aufgrund seiner Hautkrankheit	**0,782**		
Ihr Kind hatte Probleme mit der Behandlung (z. B. Behandlung zu Hause, Bandagieren, Hautpflege, usw.) aufgrund seiner Hautkrankheit	**0,656**		
Ihr Kind hatte Probleme beim An‐ und Ausziehen (Reizung der betroffenen Stellen, Schmerzen) aufgrund seiner Hautkrankheit	**0,595**	0,317	
Ihr Kind hatte Schlafprobleme aufgrund seiner Hautkrankheit	0,395	**0,357**	
Ihr Kind hatte Schmerzen aufgrund seiner Hautkrankheit		**0,930**	
Ihr Kind hat aufgrund seiner Hautkrankheit geblutet (von verletzter Haut und / oder Schleimhaut)		**0,844**	
Ihr Kind hatte Juckreiz und hat sich aufgrund seiner Hautkrankheit gekratzt		**0,726**	
Die Fragen anderer Personen nach der Hautkrankheit Ihres Kindes haben Ihr Kind verärgert			**0,752**
Ihr Kind war müde oder erschöpft aufgrund seiner Hautkrankheit			**0,748**
Es gab Zurückweisungen von Anderen aufgrund seiner Hautkrankheit			**0,733**
Ihr Kind hatte Stimmungsveränderungen aufgrund seiner Hautkrankheit	0,339		**0,539**
Ihr Kind hat sich aufgrund seiner Hautkrankheit anders gefühlt als Gleichaltrige	0,391		**0,503**
**Erklärte Gesamtvarianz (in %)**	48,8	12,9	10,2
**Cronbachs Alpha**	0,920	0,868	0,854

Faktor 1: “Alltagsaktivitäten”, Faktor 2: “Symptome”, Faktor 3: “ Beziehungen zu anderen”.

### Reliabilität

Der Cronbachs Alpha des InToDermQoL betrug 0,912 für die Altersgruppe < 1 Jahr, 0,904 für die Altersgruppe 1–2 Jahre und 0,922 für die Altersgruppe 3–4 Jahre. Die Omega‐Werte nach McDonald waren ähnlich (< 1 Jahr: ω = 0,921; 1–2 Jahre: ω = 0,902; 3–4 Jahre: ω = 0,922), was auf eine optimale interne Konsistenz in allen Altersgruppen hinweist. Einundfünfzig Familien (34,5 %) füllten den zweiten Fragebogen (T1) mit M = 17,4 Tagen (SD = 10,3; Bereich: 5–66) zwischen den beiden Messungen aus. Tabelle 5 zeigt die Test‐Retest‐Reliabilität (ICC), SEM und MDC pro Altersgruppe. Alle ICCs lagen über 0,70, was auf eine gute Test‐Retest‐Reliabilität über einen Zeitraum von zwei Wochen hinweist, mit Ausnahme der 1‐ bis 2‐jährigen Kinder (ICC_3,1_ = 0,430). Die SEM lagen zwischen 3,77 und 5,61 Punkten; die MDC_individuell_‐Werte lagen zwischen 8,89 und 15,56 Punkten.

**TABELLE 5:** Intraklassenkorrelationen (ICC), Standardmessfehler (SEM) und minimal nachweisbare Veränderung (MDC) nach Altersgruppe.

Altersgruppe	n (Retest)	ICC	95 %‐KI	SEM	MDC_individuell_	MDC_Gruppe_
< 1 Jahr	11	0,791^**^	0,285–0,942	3,77	10,46	3,15
1–2 Jahre	27	0,430^*^	−0,122–0,723	5,61	15,56	4,69
3–4 Jahre	13	0,814^**^	−0,106–0,955	3,21	8,88	2,68

*Abk*.: ICC, Intraklassenkorrelationskoeffizient; KI, Konfidenzintervall; SEM, Standardmessfehler; MDC_individuell_, minimal nachweisbare Veränderung (individuell); MDC_Gruppe_, minimal nachweisbare Veränderung (Gruppe)

* p < 0,05

** p < 0,01

### Konstruktvalidität

Alle Korrelationen zwischen den drei InToDermQoL‐Versionen und IDQOL (alle r > 0,7; für Kinder mit atopischer Dermatitis), SCORAD (0,1–0,57) und den jeweiligen Altersversionen von PedsQL^TM^ (alle r < –0,4) lagen in der erwarteten Richtung und Größenordnung (Tabelle 6). Hinsichtlich der Validität bekannter Gruppen wurden bei den schwer betroffenen Kindern im Vergleich zu den Kindern ohne oder mit leichter Belastung (GCA < 2) in allen Altersgruppen höhere InToDermQoL‐Werte festgestellt. Der Einfluss des GCA‐Wertes auf den InToDermQoL war jedoch nur bei 1‐ bis 2‐jährigen Kindern signifikant (Tabelle 7; Details siehe Tabelle S2 im Online‐Supplement). Insgesamt wurden ≥ 75 % der A‐priori‐Hypothesen bestätigt, was die Konstruktvalidität des InToDermQoL stützt.

**TABELLE 6:** Korrelationen der InToDermQoL‐Werte mit anderen Messgrößen der gesundheitsbezogenen Lebensqualität und dem Schweregrad der Erkrankung.

		InToDermQoL Altersgruppe			Hypothesen	Hypothese bestätigt?
		< 1 Jahr	1–2 Jahre	3–4 Jahre		
PedsQL^TM^	0–12 Monate	−0,628^**^			InToDermQoL und PedsQL^TM^ zeigen eine moderate negative Korrelation	Ja
	13–24 Monate		−0,415^*^			Ja
	25–48 Monate		−0,666^**^	−0,404^*^		Ja
IDQOL		0,732^**^	0,772^**^	0,878^**^	InToDermQoL und IDQOL zeigen eine moderate bis hohe positive Korrelation	Ja
SCORAD		0,109	0,565^**^	0,150	InToDermQoL und SCORAD zeigen eine geringe bis moderate positive Korrelation	Ja

* p < 0,05 (zweiseitig)

** p < 0,01 (zweiseitig)

**TABELLE 7:** Ergebnisse der ANOVAs zur Prüfung der Validität bekannter Gruppen des InToDermQoL.

Altersgruppe	InToDermQoL Wert		F‐Wert	p‐Wert	η_p_^2^	Hypothese	Hypothese bestätigt?
	GCA < 2	GCA ≥ 2					
< 1 Jahr	7,47 (± 6,97)	9,26 (± 7,49)	F(1,42) = 0,628	0,433	0,015	Kinder mit mittelschwerem bis schwerem Erkrankungsgrad (GCA ≥ 2) haben höhere InToDermQoL‐Werte als Kinder ohne oder mit leichtem Erkrankungsgrad (GCA < 2)	Nein
1–2 Jahre	9,08 (± 7,02)	14,65 (± 8,80)	F(1,66) = 8,407	0,005^*^	0,113		Ja
3–4 Jahre	13,06 (± 8,30)	16,79 (± 10,17)	F(1,34) = 1,432	0,240	0,040		Nein

* p < 0,01

## DISKUSSION

Die Hauptziele dieses Projekts bestanden darin, die deutsche Pilotversion des InToDermQoL, des ersten hautgenerischen HRQoL‐Proxy‐Instruments für Kleinkinder unter vier Jahren, zu überarbeiten und seine psychometrischen Eigenschaften in drei verschiedenen Altersgruppen zu evaluieren.

Insgesamt ergab die Analyse der Item‐Charakteristika und der Gesamtleistung der Skala positive Ergebnisse. Die Verteilung der Items über die verschiedenen Altersgruppen war im Allgemeinen akzeptabel, mit nur einer Ausnahme. In der jüngsten Altersgruppe (Kinder im Alter von 0 bis 12 Monaten) wurde ein Bodeneffekt beobachtet, der sich darin äußerte, dass ein erheblicher Teil (27,3 %) der Antworten in das unterste Viertel der Skala fiel. Dies könnte darauf hindeuten, dass die Items nicht sensibel genug sind, um Unterschiede in der HRQoL dieser Altersgruppe zu erfassen. Dennoch wurden keine weiteren signifikanten Probleme im Zusammenhang mit der Itemverteilung festgestellt.

Die explorative Faktorenanalyse lieferte Ergebnisse, die weitgehend mit den bei der Instrumentenentwicklung vorgeschlagenen Domänen übereinstimmen:^12^ Für die Altersgruppe 1–2 Jahre sind zwei Faktoren „Symptome“ und „Alltagsaktivitäten“ plausibel, während die Altersgruppe 3–4 Jahre einen dritten Faktor „Beziehungen zu anderen“ aufweist. Abweichend von den vorgeschlagenen zwei Domänen deuten die aktuellen Daten darauf hin, dass der InToDermQoL_< 1 Jahr_ eindimensional ist. In den ersten Monaten kann es für Bezugspersonen besonders schwierig sein, die Reaktionen ihres Kindes auf alltägliche Aktivitäten zu interpretieren, sodass es schwierig ist, zwischen symptombedingtem Leid und solchem aus anderen Ursachen zu unterscheiden. Wenn sich die Kommunikationsfähigkeiten des Kindes verbessern, kann es für die Bezugspersonen einfacher werden, diese Ursachen zu unterscheiden, sodass sich zwei separate Faktoren des InToDermQoL herausbilden. Mit zunehmendem Alter des Kindes tragen noch mehr Faktoren zu seiner HRQoL bei. In sehr jungen Jahren finden die sozialen Interaktionen des Kindes überwiegend innerhalb der Kernfamilie statt, die wahrscheinlich über die Hauterkrankung Bescheid weiß. Wenn das Kind jedoch in den Kindergarten kommt und beginnt, mit Gleichaltrigen zu interagieren, kann es mit den Reaktionen anderer Menschen auf seine Hauterkrankung konfrontiert werden. Darüber hinaus entwickeln Kinder erst im Alter von etwa 15 bis 18 Monaten ein Selbstbewusstsein,^33^ und beginnen ab etwa 24 Monaten, körperliche Unterschiede wahrzunehmen.^34^ Folglich beginnen auch die visuellen und sozialen Aspekte der Hauterkrankung eine Rolle für die HRQoL zu spielen. Es ist jedoch wichtig zu betonen, dass diese Ergebnisse aus einer EFA stammen und durch eine CFA mit größeren Datensätzen bestätigt werden müssen. Dennoch ist dies der erste Schritt, die Faktorstruktur des InToDermQoL zu testen, der wichtige Hypothesen für zukünftige Validierungen geliefert hat.

Die interne Konsistenz der drei Altersversionen war ausgezeichnet, und durch das Entfernen von Items aus dem Fragebogen oder den Skalen würden keine signifikanten Verbesserungen erzielt werden. Dies deutet darauf hin, dass die Items gut ausgewählt und nicht redundant sind und einen sinnvollen Beitrag zur Gesamtbewertung der HRQoL bei Kindern mit Hauterkrankungen leisten. Die Test‐Retest‐Reliabilität über einen Zeitraum von zwei Wochen war für die jüngste und älteste Altersgruppe gut. Die Reliabilität für die Gruppe der 1‐ bis 2‐Jährigen war jedoch schwach. Diese Instabilität der QoL‐Bewertungen könnte durch die raschen Entwicklungsveränderungen in dieser Altersgruppe erklärt werden. Der Zeitabstand von 2 Wochen wurde gewählt, um Erinnerungsverzerrungen zu vermeiden, wie dies für die Bewertung der Test‐Retest‐Reliabilität allgemein empfohlen wird.^35^, ^36^ Allerdings ändern sich Verhalten und Kommunikationsfähigkeiten kleiner Kinder häufig schnell, was sich auch auf die Wahrnehmung ihrer Eltern auswirkt. Im zweiten Lebensjahr entwickeln und verfeinern sich wichtige grob‐ und feinmotorische Fähigkeiten (zum Beispiel Laufen, Hand‐Augen‐Koordination) sowie kognitive und erste Sprachfähigkeiten.^37^ Darüber hinaus ist es in Deutschland üblich, dass Kinder im Alter von etwa 12 Monaten mit dem Ende der Elternzeit in den Kindergarten kommen. Diese Erweiterung des Umfelds kann durch den sozialen Vergleich mit anderen Kindern oder den Erfahrungsaustausch mit anderen Eltern rasche Veränderungen sowohl im Verhalten des Kindes als auch in der Wahrnehmung der Eltern bewirken. Daher sind weitere Studien erforderlich, um zu bestätigen, ob die schwache Test‐Retest‐Reliabilität in dieser spezifischen Altersgruppe eine psychometrische Schwäche des Fragebogens ist oder tatsächliche Veränderungen im gemessenen Konstrukt widerspiegelt.

Konvergente Validitätsanalysen zeigten Korrelationen in erwarteter Richtung und Größe mit der allgemeinen HRQoL (PedsQL^TM^) sowie mit der atopischen Dermatitis‐bezogenen QoL (IDQOL) und dem Schweregrad der Erkrankung (SCORAD) bei Kindern mit atopischer Dermatitis. Dies deutet darauf hin, dass der InToDermQoL das Konstrukt „Hauterkrankungs‐bezogene Lebensqualität” bei Kleinkindern, wie sie von ihren Bezugspersonen angegeben wird, effektiv erfasst. Ein besonders bemerkenswertes Ergebnis war die starke positive Korrelation zwischen den InToDermQoL‐Werten für 1‐ bis 2‐Jährige und dem Schweregrad der Erkrankung, gemessen anhand des SCORAD. Dies könnte darauf hindeuten, dass der Schweregrad der Erkrankung eine besonders wichtige Rolle für die HRQoL in dieser Altersgruppe spielt. Dies könnte zudem die relativ geringere Test‐Retest‐Reliabilität bei 1‐ bis 2‐Jährigen erklären, da Veränderungen des Schweregrads der Erkrankung zu größeren Schwankungen der von den Bezugspersonen angegebenen HRQoL führen können. Hinsichtlich der Validität bekannter Gruppen zeigte die Skala keine gute Unterscheidungsfähigkeit zwischen leicht und mittelschwer bis schwer betroffenen Fällen, was durch die relativ kleine Stichprobengröße erklärt werden könnte. Insgesamt wurde die Konstruktvalidität dennoch bestätigt.

Obwohl die Ergebnisse dieser Studie vielversprechend sind, sind einige Einschränkungen zu berücksichtigen. Erstens wurde, wie aufgrund früherer Studien und der allgemein hohen Prävalenz von atopischer Dermatitis zu erwarten war,^14^, ^15^ bei der Mehrheit der in die Studie einbezogenen Kinder diese Erkrankung diagnostiziert. Dies schränkt die Verallgemeinerbarkeit der Ergebnisse auf Kinder mit anderen Hauterkrankungen ein. Zukünftige Studien sollten darauf abzielen, eine vielfältigere Stichprobe von Kindern mit verschiedenen Hauterkrankungen zu rekrutieren, um eine breitere Anwendbarkeit des Instruments sicherzustellen. Zweitens waren die meisten Befragten Mütter. Obwohl frühere Untersuchungen keine signifikanten Unterschiede zwischen Müttern und Vätern bei der Bewertung der HRQoL ihrer Kinder anhand des IDQOL gezeigt haben,^38^, ^39^ muss die Interrater‐Reliabilität des InToDermQoL noch getestet werden. Zukünftige Studien sollten darauf abzielen, nach Möglichkeit Bewertungen von beiden Elternteilen sowie von anderen Bezugspersonen zu sammeln, um zu beurteilen, ob verschiedene Bezugspersonen die HRQoL von Kindern konsistent bewerten. Darüber hinaus ist eine zukünftige Interventionsstudie erforderlich, um den minimal klinisch relevanten Unterschied der deutschen Version des InToDermQoL zu ermitteln und seine Responsivität auf Veränderungen des Gesundheitszustands von Kindern zu testen, was für den Einsatz in der klinischen Praxis und Forschung von entscheidender Bedeutung ist.

Der InToDermQoL ist das erste hautgenerische HRQoL‐Instrument für Kinder unter 4 Jahren, das nun in deutscher Sprache verfügbar ist. In Übereinstimmung mit anderen Sprachversionen zeigte die deutsche Version in der vorliegenden Studie eine gute Reliabilität und Validität über alle Altersgruppen hinweg. Das Instrument hat ein erhebliches Potenzial für den Einsatz in Forschung und Klinik, da es wertvolle Einblicke in die Auswirkungen von Hauterkrankungen auf die HRQoL von Kleinkindern und ihren Familien liefert. Damit hat das Instrument das Potenzial, zu einem stärker personenzentrierten Ansatz in der Gesundheitsversorgung beizutragen, wie er von der WHO,^11^ und anderen,^40^ gefordert wird. In diesem Sinne kann ein besseres Verständnis darüber, wie Hauterkrankungen diese gefährdete Bevölkerungsgruppe von Säuglingen und Kleinkindern beeinträchtigen, letztlich zu einer verbesserten medizinischen Versorgung und besseren Langzeitergebnissen für Kinder mit Hauterkrankungen beitragen.

## DANKSAGUNG

Die Autoren danken allen Familien für ihre Teilnahme, Pavel Chernyshov für seine Unterstützung und Beratung bei der Konzeption der Studie und dem Wissenschaftskommunikationsteam des IVDP, insbesondere Paula Willer, für die Bearbeitung des Artikels.

Open access Veröffentlichung ermöglicht und organisiert durch Projekt DEAL.

## FINANZIERUNG

Diese Studie wurde von Eli Lilly Germany finanziert.

## INTERESSENKONFLIKT

M.A. war als Berater, Dozent und Forscher tätig und/oder hat institutionelle Forschungsförderungen von Unternehmen erhalten, die Medikamente für Hauterkrankungen bei Kindern herstellen, einschließlich AbbVie, Almirall, Amgen, Bayer, Beiersdorf, Eli Lilly, Galderma, Incyte, Janssen, LEO, L'Oréal, Novartis, Pfizer, Regeneron, Sanofi und Viatris. P.S. war als Berater, Dozent und Forscher tätig und/oder hat institutionelle Forschungsförderungen von Unternehmen erhalten, die Medikamente für Hauterkrankungen bei Kindern herstellen, einschließlich AbbVie, Almirall, Amgen, Beiersdorf, Biocryst, BMS, Celltrion, CSL Behring, Eli Lilly, Galderma, Incyte, Janssen, Leo Pharma, Regeneron, Sanofi, L'Oréal, Klinge, Pierre Fabre, Novartis, Pfizer, Stadapharma, Takeda, Unna Academy und UCB Pharma. R.F.‐H. war als Berater und Dozent für die Unternehmen Allergika, Infectopharm, La Roche Posay, LEO, Novartis, Pierre Fabre, Regeneron, Sanofi und Viatris tätig. J.T., N.d.S.B., H.O., S.H., M.B. und R.S. erklären, dass kein Interessenkonflikt besteht.

## Supporting information



Supplementary information

Supplementary information

## References

[1] Augustin M , Radtke MA , Glaeske G , et al. Epidemiology and comorbidity in children with psoriasis and atopic eczema. Dermatology. 2015;231:35‐40. 25966818 10.1159/000381913

[2] Basra MKA , Finlay AY . The family impact of skin diseases: the Greater Patient concept. Br J Dermatol. 2007;156:929‐937. 17381458 10.1111/j.1365-2133.2007.07794.x

[3] Pauli‐Pott U , Darui A , Beckmann D . Infants with atopic dermatitis: maternal hopelessness, child‐rearing attitudes and perceived infant temperament. Psychother Psychosom. 1999;68:39‐45. 9873241 10.1159/000012309

[4] Kelly KA , Balogh EA , Kaplan SG , et al. Skin disease in children: Effects on quality of life, stigmatization, bullying, and suicide risk in pediatric acne, atopic dermatitis, and psoriasis patients. Children. 2021;8:1057. 34828770 10.3390/children8111057 PMC8619705

[5] Vivar KL , Kruse L . The impact of pediatric skin disease on self‐esteem. Int J Womens Dermatol. 2018;4:27‐31. 29872673 10.1016/j.ijwd.2017.11.002 PMC5986112

[6] Ablett K , Thompson AR . Parental, child, and adolescent experience of chronic skin conditions: A meta‐ethnography and review of the qualitative literature. Body Image. 2016;19:175‐185. 27768987 10.1016/j.bodyim.2016.10.001

[7] Mitchell AE . Bidirectional relationships between psychological health and dermatological conditions in children. Psychol Res Behav Manag. 2018;11:289‐298. 30104911 10.2147/PRBM.S117583 PMC6074762

[8] Jager MEA de , van de Kerkhof PCM , Jong EMGJ de , et al. A cross‐sectional study using the Children's Dermatology Life Quality Index (CDLQI) in childhood psoriasis: Negative effect on quality of life and moderate correlation of CDLQI with severity scores. Br J Dermatol. 2010;163:1099‐1101. 20716218 10.1111/j.1365-2133.2010.09993.x

[9] Khoury LR , Skov L , Møller T . Facing the dilemma of patient‐centred psoriasis care: A qualitative study identifying patient needs in dermatological outpatient clinics. Br J Dermatol. 2017;177:436‐444. 28032892 10.1111/bjd.15292

[10] Stephan B , Sommer R , Augustin M , et al. Need for individualized counseling regarding psoriasis systemic therapy in women of childbearing age: analysis of the PsoFem study at the University Medical Center Hamburg. Int J Womens Dermatol. 2024;10:e187. 39555230 10.1097/JW9.0000000000000187 PMC11567713

[11] WHO . Global report on psoriasis 2016, World health organization, Geneva, 2016.

[12] Chernyshov PV , Boffa MJ , Corso R , et al. Creation and pilot test results of the dermatology‐specific proxy instrument: the Infants and Toddlers Dermatology Quality of Life. J Eur Acad Dermatol Venereol. 2018;32:2288‐2294. 30169902 10.1111/jdv.15229

[13] Ravens‐Sieberer U , Schmidt S , Gosch A , et al. Measuring subjective health in children and adolescents: Results of the European KIDSCREEN/DISABKIDS Project. Psychosoc Med. 2007;4:Doc08. 19742297 PMC2736532

[14] Chernyshov PV , Sampogna F , Pustišek N , et al. Validation of the dermatology‐specific proxy instrument the Infants and Toddlers Dermatology Quality of Life. J Eur Acad Dermatol Venereol. 2019;33:1405‐1411. 30767284 10.1111/jdv.15496

[15] Chernyshov PV , Marron SE , Koumaki D , et al. Responsiveness and minimal clinically important difference of the Infants and Toddlers Dermatology Quality of Life questionnaire. Dermatol Ther (Heidelb). 2023;13:2879‐2893. 37731087 10.1007/s13555-023-01022-x PMC10613170

[16] Chernyshov PV , Marron SE , Boffa MJ , et al. Sensitivity to treatment and score bands of the Infants and Toddlers Dermatology Quality of Life questionnaire. JAAD Int. 2023;10:61‐67. 36688100 10.1016/j.jdin.2022.11.009 PMC9850170

[17] US Food and Drug Administration . Guidance for industry: patient‐reported outcome measures: Use in medical product development to support labeling claims, 2009. 10.1186/1477-7525-4-79 PMC1629006 17034633

[18] Drotar D . Validating measures of pediatric health status, functional status, and health‐related quality of life: key methodological challenges and strategies. Ambul Pediatr. 2004;4:358‐364. 15264947 10.1367/A03-101R.1

[19] Guyatt GH , Feeny DH , Patrick DL . Measuring health‐related quality of life. Ann Intern Med. 1993;118:622‐629. 8452328 10.7326/0003-4819-118-8-199304150-00009

[20] Rullo V , Segato A , Kirsh A , et al. Severity scoring of atopic dermatitis: A comparison of two scoring systems. Allergologia et Immunopathologia. 2008;36:205‐211. 18928687

[21] Lewis‐Jones MS , Finlay AY , Dykes PJ . The Infants' Dermatitis Quality of Life Index. Br J Dermatol. 2001;144:104‐110. 11167690 10.1046/j.1365-2133.2001.03960.x

[22] Varni JW , Limbers CA , Neighbors K , et al. The PedsQL™ Infant Scales: Feasibility, internal consistency reliability, and validity in healthy and ill infants. Qual Life Res. 2011;20:45‐55. 20730626 10.1007/s11136-010-9730-5

[23] Varni JW , Burwinkle TM , Seid M , et al. The PedsQL™* 4.0 as a pediatric population health measure: Feasibility, reliability, and validity. Ambul Pediatr. 2003;3:329‐341. 14616041 10.1367/1539-4409(2003)003<0329:tpaapp>2.0.co;2

[24] Niewerth M , Minden K , Biedermann T , et al. Pediatric Quality of Life Inventory: Evaluierung eines deutschen Messinstrumentes zur Beurteilung der gesundheitsbezogenen Lebensqualität bei Kindern und Jugendlichen mit juveniler idiopathischer Arthritis. Akt Rheumatol. 2003;28: doi:10.1055/s-2003-45071.

[25] Kaiser HF . An index of factorial simplicity. Psychometrika. 1974;39:31‐36.

[26] Thurstone LL . Multiple factor analysis, University of Chicago, Chicago, IL, 1947.

[27] Koo TK , Li MY . A guideline of selecting and reporting intraclass correlation coefficients for reliability research. J Chiropr Med. 2016;15:155‐163. 27330520 10.1016/j.jcm.2016.02.012 PMC4913118

[28] Vet HCW de , Terwee CB , Mokkink LB , et al. Measurement in Medicine, Cambridge University Press, 2011.

[29] Geerinck A , Alekna V , Beaudart C , et al. Standard error of measurement and smallest detectable change of the Sarcopenia Quality of Life (SarQoL) questionnaire: An analysis of subjects from 9 validation studies. PLoS One. 2019;14:e0216065. 31034498 10.1371/journal.pone.0216065 PMC6488089

[30] Kovacs FM , Abraira V , Royuela A , et al. Minimum detectable and minimal clinically important changes for pain in patients with nonspecific neck pain. BMC Musculoskelet Disord. 2008;9:43. 18402665 10.1186/1471-2474-9-43 PMC2375888

[31] Fleiss JL , Cohen J . The equivalence of weighted kappa and the intraclass correlation coefficient as measures of reliability. Educ Psychol Meas. 1973;33:613‐619.

[32] Tabachnick BG , Fidell LS , Ullman JB . Using multivariate statistics, Pearson, Boston, 2019.

[33] Brownell CA , Zerwas S , Ramani GB . "So big": The development of body self‐awareness in toddlers. Child Dev. 2007;78:1426‐1440. 17883440 10.1111/j.1467-8624.2007.01075.x PMC3351035

[34] Slaughter V , Heron M . Origins and early development of human body knowledge. Monogr Soc Res Child Dev. 2004;69:vii, 1‐102. 15317497 10.1111/j.0037-976X.2004.00286.x

[35] Streiner DL , Norman GR . Health Measurement Scales: A practical guide to their development and use. 4th ed., Oxford University Press, New York, 2008.

[36] Prinsen CAC , Mokkink LB , Bouter LM , et al. COSMIN guideline for systematic reviews of patient‐reported outcome measures. Qual Life Res. 2018;27:1147‐1157. 29435801 10.1007/s11136-018-1798-3 PMC5891568

[37] Courage ML , Howe ML . From infant to child: The dynamics of cognitive change in the second year of life. Psychol Bull. 2002;128:250‐277. 11931519 10.1037/0033-2909.128.2.250

[38] Chernyshov PV . May the gender of a parent influence assessment of health‐related quality of life, family impact and severity of atopic dermatitis in children? Pediatr Dermatol. 2009;26:99‐100. 19250422 10.1111/j.1525-1470.2008.00834.x

[39] Holm EA , Esmann S , Jemec GBE . Parent gender and assessment of infant life quality. J Eur Acad Dermatol Venereol. 2006;20:274‐276. 16503886 10.1111/j.1468-3083.2006.01421.x

[40] Sommer R , Mrowietz U , Du Gaarn Jardin K , et al. Implementing well‐being in the management of psoriasis: An expert recommendation. J Eur Acad Dermatol Venereol. 2024;38:302‐310. 37822008 10.1111/jdv.19567

