# Supplementary material for: Validierung der deutschen Version des Fragebogens zur dermatologischen Lebensqualität von Säuglingen und Kleinkindern (InToDermQoL)
Source: J Dtsch Dermatol Ges. 2026 Sep 8;24(9):1251–63. [Article in German] doi: 10.1111/ddg.16008_g (PMC13551125; doi:10.1111/ddg.16008_g)
Supplement: Supplementary file 1 [file DDG-24-1251-s001.docx]

**Anhang 1**

**Tabelle S1.** Beschreibende Statistiken und interne Konsistenz (Cronbachs Alpha) von IDQOL und PedsQL^TM^ für die Gesamtstichprobe und Unterschiede zwischen den Altersgruppen.

| **Fragebogen** | **Skala** | **Altersgruppe** | | **M (SD)** | **Bereich** | **Unterschiede zwischen den Gruppen** | **Cronbachs Alpha** |
| --- | --- | --- | --- | --- | --- | --- | --- |
| IDQOL |  | Gesamt | | 8,58 (5,89) | 0 – 28 |  | 0,880 |
|  |  | < 1 Jahr | | 8,36 (5,09) | 0 – 19 | F (2,221, 25,16) = 0,063, p = 0,939 |  |
|  |  | 1 – 2 Jahre | | 8,56 (6,22) | 1 – 28 |  |  |
|  |  | 3 – 4 Jahre | | 8,86 (6,02) | 1 – 24 |  |  |
| PedsQL^TM^ | Gesamt | < 1 Jahr | | 79,74 (10,66) | 54,85 – 99,31 |  | 0,877 |
|  | körperlich |  |  | 79,52 (17,49) | 41,67 – 100 |  |  |
|  | Symptome |  |  | 83,73 (14,62) | 17,50 – 100 |  |  |
|  | emotional |  |  | 72,83 (14,02) | 45,83 – 100 |  |  |
|  | sozial |  |  | 89,18 (14,99) | 50 – 100 |  |  |
|  | kognitiv |  |  | 83,33 (18,38) | 37,50 – 100 |  |  |
|  | Gesamt | 1 – 2 Jahre* | 13 – 24 | 85,10 (7,54) | 70 – 100 |  | 0.872 |
|  |  |  | 25 – 36 | 84,45 (8,67) | 58,75 – 100 |  |  |
|  | körperlich |  | 13 – 24 | 84,47 (13,57) | 61,11 – 100 |  |  |
|  |  |  | 25 – 36 | 87,42 (10,49) | 56,25 – 100 |  |  |
|  | Symptome |  | 13 – 24 | 92,15 (5,89) | 82,50 – 100 |  |  |
|  | emotional |  | 13 – 24 | 73,56 (13,27) | 54,17 – 100 |  |  |
|  |  |  | 25 – 36 | 71,35 (14,09) | 45 – 100 |  |  |
|  | sozial |  | 13 – 24 | 93,20 (9,23) | 65 – 100 |  |  |
|  |  |  | 25 – 36 | 91,45 (10,77) | 60 – 100 |  |  |
|  | kognitiv |  | 13 – 24 | 89,31 (13,94) | 50 – 100 |  |  |
|  | Kindergarten |  | 25 – 36 | 88,11 (16,03) | 41,67 – 100 |  |  |
|  | Gesamt | 3 – 4 Jahre | | 85,13 (13,51) | 51,19 – 100 |  | 0,837 |
|  | körperlich |  |  | 88,28 (16,12) | 40,63 – 100 |  |  |
|  | emotional |  |  | 77,92 (17,66) | 35 – 100 |  |  |
|  | sozial |  |  | 91 (13,66) | 55 – 100 |  |  |
|  | Schule |  |  | 85,35 (18,75) | 50 – 100 |  |  |

*PedsQL^TM^ verwendet andere Altersgruppen als der InToDermQoL, sodass Eltern von Kindern im Alter von 1 bis 2 Jahren je nach dem genauen Alter ihres Kindes entweder die PedsQL^TM^-Version für Kinder im Alter von 12 bis 24 Monaten oder die Version für Kinder im Alter von 25 bis 48 Monaten ausfüllen mussten.
