# Supplementary material for: Validierung der deutschen Version des Fragebogens zur dermatologischen Lebensqualität von Säuglingen und Kleinkindern (InToDermQoL)
Source: J Dtsch Dermatol Ges. 2026 Sep 8;24(9):1251–63. [Article in German] doi: 10.1111/ddg.16008_g (PMC13551125; doi:10.1111/ddg.16008_g)
Supplement: Supplementary file 2 [file DDG-24-1251-s002.docx]

**Anhang 2**

*Detaillierte Ergebnisse*

*Strukturelle Validität.* Der Bartlett-Test auf Sphärizität ergab, dass die Korrelationsmatrix nicht zufällig war (< 1 Jahr: χ^2^ (45) = 290,95, p < 0,001; 1–2 Jahre: χ^2^ (66) = 453,31, p < 0,001; 3–4 Jahre: χ^2^ (105) = 286,69, p < 0,001). Die KMO-Statistik lag über dem empfohlenen Schwellenwert von 0,6^25^ (< 1 Jahr: 0,807; 1 – 2 Jahre: 0,823; 3 – 4 Jahre: 0,690). Daher wurde befunden, dass die Daten für eine Faktorenanalyse geeignet waren.

In der jüngsten Altersgruppe deutete das Scree-Plot auf die Beibehaltung von zwei Faktoren hin. Acht Items luden auf Faktor 1 und zwei Items auf Faktor 2, von denen eines auch auf Faktor 1 quergeladen war (0,32). Faktor 1 erklärte 56,7 % der Gesamtvarianz und hatte einen Cronbachs Alpha-Wert von 0,921; Faktor 2 erklärte 13,2 % der Gesamtvarianz und hatte einen Cronbachs Alpha-Wert von 0,763. Die beiden Faktoren korrelierten mit 0,324 und zeigten eine relativ ähnliche Muster- und Strukturmatrix. Alternativ wurde eine 3-Faktoren-Lösung untersucht, bei der sechs Items auf Faktor 1 luden, von denen zwei auch auf Faktor 3 querluden (beide > 0,34), und jeweils zwei Items auf die Faktoren 2 und 3 luden. Faktor 1 machte 56,7 % der Gesamtvarianz aus und hatte einen Cronbachs Alpha-Wert von 0,929. Faktor 2 machte 13,2 % der Gesamtvarianz aus und hatte einen Cronbachs Alpha-Wert von 0,763. Faktor 3 machte 9,5 % der Gesamtvarianz aus und hatte einen Cronbachs Alpha-Wert von 0,729. Die Faktoren 1 und 2 korrelierten mit 0,484, die Faktoren 1 und 3 mit 0,550 und die Faktoren 2 und 3 mit 0,209, was eine gewisse Divergenz zwischen Muster- und Strukturmatrix widerspiegelt. Keine dieser Lösungen erfüllte alle vier Kriterien, da in beiden Fällen mindestens ein Faktor mit weniger als drei Variablen geladen war. Eine Ein-Faktor-Lösung mit allen 10 Items erfüllte das Kriterium, > 60 % der Gesamtvarianz zu erklären, nicht. Nach Löschen von Item 8 („Essstörungen“), das als einziges in der 2-Faktor-Lösung nicht auf Faktor 1 lud, wurde dieses Kriterium jedoch erfüllt. Die eindimensionale Skala mit neun Items erklärte 60,97 % der Gesamtvarianz und hatte einen Cronbachs Alpha-Wert von 0,917. Obwohl ursprünglich zwei Domänen für diese Altersversion vorgeschlagen wurden,^12^ ist eine eindimensionale Struktur theoretisch plausibel: Da Säuglinge nur über sehr wenig Autonomie und stark eingeschränkte Fähigkeiten zur Kommunikation ihrer Bedürfnisse verfügen, kann es für Bezugspersonen besonders schwierig sein, Weinen, Rückzug von Berührungen usw. der Hauterkrankung zuzuordnen und von anderen Umständen zu unterscheiden, d. h. zwischen Reaktionen auf die Hauterkrankung und allgemeineren Stimmungen zu unterscheiden. Diese Hypothese muss in einer anderen Stichprobe mittels CFA überprüft werden.

Für die Altersgruppe 1–2 Jahre deute das Scree-Plot-Diagramm auf Beibehaltung von zwei Faktoren. Fünf Items luden auf Faktor 1 und sieben Items auf Faktor 2, von denen zwei Querladungen auf Faktor 1 aufwiesen (beide > 0,33). Faktor 1 machte 49,9 % der Gesamtvarianz aus und hatte einen Cronbachs Alpha-Wert von 0,910; Faktor 2 machte 13,8 % der Gesamtvarianz aus und hatte einen Cronbachs Alpha-Wert von 0,843. Die beiden Faktoren korrelierten mit 0,497 und zeigten eine weitgehend ähnliche Muster- und Strukturmatrix. Faktor 1 wurde als „Symptome” interpretiert, während Faktor 2 in Übereinstimmung mit den Vorschlägen bei der Instrumentenentwicklung^12^ als „Alltagsaktivitäten” bezeichnet wurde. Diese Zwei-Faktoren-Lösung (siehe Tabelle 3) wurde als die am besten geeignete strukturelle Darstellung der InToDermQoL-Version für Kinder im Alter von 1 bis 2 Jahren angesehen.

Für die Altersgruppe der 3- bis 4-Jährigen deutete das Scree-Plot auf Beibehaltung von drei Faktoren. Sieben Items luden auf Faktor 1, davon zwei mit Kreuzladung auf Faktor 2 (> 0,31), drei Items luden auf Faktor 2 und fünf Items auf Faktor 3, davon wiederum zwei mit Kreuzladung auf Faktor 1 (> 0,33). Faktor 1 erklärte 48,8 % der Gesamtvarianz und hatte einen Cronbachs Alpha-Wert von 0,919. Faktor 2 erklärte 12,9 % der Gesamtvarianz und hatte einen Cronbachs Alpha-Wert von 0,888. Faktor 3 machte 10,2 % der Gesamtvarianz aus und hatte einen Cronbachs Alpha-Wert von 0,854. Die Faktoren 1 und 2 korrelierten mit 0,367, die Faktoren 1 und 3 mit 0,497 und die Faktoren 2 und 3 mit 0,317, mit relativ ähnlicher Muster- und Strukturmatrix. In einer alternativen Zwei-Faktoren-Lösung luden zwölf Items auf Faktor 1, von denen eines eine Querladung auf Faktor 2 aufwies (0,34), und drei Items luden auf Faktor 2. Die Faktoren 1 und 2 wiesen Cronbach-Werte von α = 0,921 bzw. α = 0,888 auf, korrelierten mit 0,439 und zeigten eine weitgehend ähnliche Muster- und Strukturmatrix. Die Drei-Faktoren-Lösung (siehe Tabelle 4) wurde als die am besten geeignete strukturelle Darstellung der InToDermQoL-Version für Kinder im Alter von 3 bis 4 Jahren angesehen. In Übereinstimmung mit den vorgeschlagenen Domänen^12^ wurde Faktor 1 als „Alltagsaktivitäten ”, Faktor 2 als „Symptome” und Faktor 3 als „Beziehungen zu anderen” interpretiert. Da Item 4 („Schlafprobleme“) eine Querladung auf Faktor 2 „Symptome“ aufweist und theoretisch besser zu diesem Faktor passt (siehe Altersgruppe 1–2 Jahre), wurde Item 4 im endgültigen Modell Faktor 2 zugeordnet. Somit besteht Faktor 1 aus sechs Items mit einem Cronbachs Alpha von 0,920 und Faktor 2 aus vier Items mit einem Cronbachs Alpha von 0,868, während Faktor 3 unverändert bleibt.

*Reliabilität*. Einundfünfzig Familien (34,46 %) füllten den zweiten Fragebogen (T1) aus, wobei zwischen den beiden Messungen M = 17,40 Tage (SD = 10,26; Spannweite: 5 – 66) lagen. In zwei Fällen wurden die Fragebögen T0 und T1 nicht von denselben Eltern ausgefüllt. Es gab keine signifikanten Unterschiede zwischen den soziodemografischen und klinischen Variablen des Kindes oder dem Alter der antwortenden Betreuungsperson zwischen denjenigen, die den Fragebogen T1 ausgefüllt hatten, und denjenigen, die dies nicht getan hatten (alle p > 0,1). Anzumerken ist, dass in der jüngsten Altersgruppe der InToDermQoL-Score zu T0 bei den Säuglingen, deren Eltern T1 ausgefüllt hatten (M = 12,45, SD = 6,91), signifikant höher war als bei denen, die T1 nicht ausgefüllt hatten (M = 7,27, SD = 7,00; t (42) = 2,133, p = 0,039, 95 % KI [0,038, 1,439]). In den beiden älteren Altersgruppen unterschieden sich die InToDermQoL-Werte zwischen den Teilnehmern, die den Fragebogen vollständig ausgefüllt hatten, und denen, die dies nicht getan hatten, nicht (beide p < 0,4).

Die SEms lagen zwischen 3,77 und 5,61 Punkten, was darauf hindeutet, dass der „wahre” Wert eines Probanden mit einer Konfidenz von 68 % (± 1 SEm) zwischen -3,77/5,61 und +3,77/5,61 Punkten vom beobachteten Wert und mit einer Konfidenz von 95 % (± 2 SEm) zwischen -7,54/11,22 und +7,54/11,22 Punkten vom beobachteten Wert liegt. Die MDC_individuell_ Werte lagen zwischen 8,88 und 15,56 Punkten. Dies bedeutet, dass sich die Punktzahl eines Kindes auf dem InToDermQoL um mindestens 8,88 Punkte (auf einer Skala von 0 bis 30) für Kinder < 1 Jahr, 10,46 Punkte (auf einer Skala von 0 bis 36) bei 1- bis 2-Jährigen und 15,56 Punkte (auf einer Skala von 0 bis 45) bei 3- bis 4-Jährigen ändern müsste, damit die beobachtete Veränderung als echte Veränderung der HRQoL und nicht als potenzielles Ergebnis eines Messfehlers angesehen werden kann.

*Validität bekannter Gruppen.* Tabelle S2 zeigt die relevanten Merkmale der Untergruppen, d. h. bei unterschiedlichen Schweregraden der Erkrankung, bewertet anhand des GCA.

**Tabelle S2.** Demografische und klinische Merkmale [M (± SD)] der eingeschlossenen Kinder in verschiedenen Altersgruppen nach Schweregrad der Erkrankung (GCA).

|  |  | **< 1 Jahr** | **1 – 2 Jahre** | **3 – 4 Jahre** |
| --- | --- | --- | --- | --- |
| **GCA < 2** |  | **(n = 17)** | **(n = 37)** | **(n = 17)** |
| Geschlecht (%) | weiblich | 8 | 13 | 8 |
|  | männlich | 9 | 24 | 9 |
| Alter (in Jahren) | | 0,57 (± 0,27) | 1,87 (± 0,57) | 3,42 (± 0,50) |
| Alter bei Erstauftreten (in Jahren) | | 0,23 (± 0,19) | 0,66 (± 0,51) | 0,76 (± 0,68) |
| Therapie (%)* | keine | 1 (5,9) | 1 (2,8) | 1 (5,9) |
|  | topisch | 10 (58,8) | 33 (91,7) | 16 (94,1) |
|  | systemisch | - | 5 (13,9) | - |
|  | andere | 7 (41,2) | 2 (5,6) | 1 (5,9) |
| BSA (in %) | | 9,15 (± 11,29) | 12,74 (± 17,49) | 7,79 (± 6,18) |
| SCORAD‡ | | 25,96 (± 6,33) | 22,99 (± 13,27) | 20,15 (± 10,44) |
| **GCA ≥ 2** | | **(n = 27)** | **(n = 31)** | **(n = 19)** |
| Geschlecht (%) | weiblich | 13 | 10 | 13 |
|  | männlich | 14 | 21 | 6 |
| Alter (in Jahren) | |  | 1,97 (± 0,54) | 3,28 (± 0,45) |
| Alter bei Erstauftreten (in Jahren) | |  | 0,62 (± 0,61) | 0,93 (± 0,90) |
| Therapie (%)* | keine | 1 (3,7) | 30 (100) | - |
|  | topisch | 24 (88,9) | 28 (93,3) | 19 (100) |
|  | systemisch | 7 (25,9) | 6 (20) | - |
|  | andere | 3 (11,1) | 3 (10) | 1 (5,3) |
| BSA (in %) | | 19,28 (± 18,21) | 26,06 (± 22,33) | 26,68 (± 22,16) |
| SCORAD‡ | | 39,62 (± 14,01) | 38,24 (± 18,42) | 39,86 (± 12,91) |

GCA = Global Clinical Assessment (< 2: keine Belastung – leicht, ≥ 2: mittel – sehr schwer); BSA = Körperoberfläche; SCORAD = SCORing Atopic Dermatitis (Bewertung der atopischen Dermatitis); *einige Kinder erhielten mehr als eine Behandlungsart. ‡nur bei Kindern mit atopischer Dermatitis ausgefüllt.
